# Global Transmission and Evolution of Chikungunya Virus: Origins, Adaptive Mutations, and Intercontinental Spread of the Three Genotypes

**DOI:** 10.1155/tbed/3315650

**Published:** 2025-11-26

**Authors:** Yujia Hao, Qingmiao Fan, Fan Yu, Fei Xu, Huiling Qin, Yuge Yuan, Wenzhou Ma, Duo Zhang, Chengcheng Peng, Nan Li, Pengpeng Xiao

**Affiliations:** ^1^Wenzhou Key Laboratory for Virology and Immunology, Institute of Virology, Wenzhou University, Wenzhou, Zhejiang, China; ^2^College of Veterinary Medicine, Jilin University, Changchun, Jilin, China

**Keywords:** CHIKV, evolution, mutation, positive selection, transmission dynamics

## Abstract

Chikungunya virus (CHIKV) is an arthropod-borne virus that has caused several major outbreaks around the world and is becoming increasingly harmful. Although significant progress has been made in understanding the global epidemiology and transmission of CHIKV, a systematic description of the transmission history of its three genotypes is still lacking. To address this gap, this study integrates multiple bioinformatics approaches to explore their origin, evolution, and transmission dynamics. We analyzed publicly available CHIKV genomes from NCBI to elucidate the genetic evolution and transmission potential of these genotypes. Phylogeographic and molecular evolutionary analyses showed that the West African (WA) genotype originated in Nigeria and spread exclusively within Africa; the Eastern/Central/South African (ECSA) genotype originated in Tanzania and spread globally; and the Asian genotype originated in Thailand, spread throughout Asia, Oceania, and the Americas, exhibiting the highest evolutionary rate among the three genotypes. We also identified 15 positively selected sites and 10 nonconservative mutation sites with altered hydrophobicity across CHIKV proteins, all of which need further investigation into their effects on viral protein function. The data from this study are important for understanding the transmission history of the three genotypes of CHIKV, providing new targets for CHIKV antiviral therapy and ideas for developing effective prevention and control measures in the future.

## 1. Introduction

Chikungunya virus (CHIKV) is a positive-stranded RNA virus of *Alphavirus* transmitted by *Aedes* with a global distribution. The genomic structure of CHIKV contains two open reading frames (ORFs) encoding nonstructural proteins (NSP1, NSP2, NSP3, and NSP4) and structural proteins (capsid protein [C], E3, E2, 6K, and E1) [1]. Although CHIKV infection may be asymptomatic, symptomatic patients may develop chikungunya fever (CHIKF), which is characterized by sudden onset of high fever (39–40°C), maculopapular rash, and persistent joint pain with hyperviremia and antigenemia. The course of the disease usually lasts 1–3 weeks, and some patients may develop chronic arthritis that lasts for several months and up to several years [2, 3]. Since its emergence in Africa, CHIKV has spread rapidly, causing more than 10 million cases in more than 125 countries or territories over the past two decades and posing a global public health threat [4].

CHIKV originated in Africa and was first isolated in Tanzania in 1953 [5, 6]. Phylogenetic analysis identified three major lineages of CHIKV: West African (WA), Eastern/Central/South African (ECSA), and Asian genotypes [7]. Since its discovery, CHIKV has been spreading and causing sporadic outbreaks in sub-Saharan Africa [8]. After emerging in Kenya in 2004, the ECSA strain of CHIKV spread to the Indian Ocean islands, triggering an unprecedented outbreak, notably on Réunion Island. It subsequently reached Asia and India, and eventually caused indigenous transmission in European countries, such as Italy and France [9–12]. Another major outbreak occurred in December 2013, when the Asian genotype of CHIKV first emerged on the Caribbean island of Saint Martin and spread to 22 countries across the Caribbean, Central, and South America within just 9 months, resulting in hundreds of thousands of cases [13]. In 2014, the ECSA was reported in northeastern Brazil, where the strain is still spreading today as the most prevalent strain [14].

CHIKV is transmitted mainly through the bites of infected mosquitoes. Depending on the region, transmission occurs via the sylvatic cycle and the urban cycle. In Africa, CHIKV is mainly transmitted in the sylvatic cycle, involving wild non-human primates and forest-dwelling *Aedes* [15]. Occasionally, the virus jumps to populations living near forested areas. Similar zoonotic transmission has also been reported in Asia [16]. The urban cycle is the transmission of CHIKV between humans and mosquitoes in cities, with *Aedes aegypti* (*Ae. aegypti*) and *Aedes albopictus* (*Ae. albopictus*) being the main vectors in this cycle. The urban cycle of CHIKV has been associated with several large CHIKV epidemics on different continents, including Asia, Europe, and North America [17]. The distributions of *Ae. aegypti* and *Ae. albopictus* are projected to expand due to environmental adaptation and increasing human mobility, raising the likelihood of CHIKV spread to new regions and underscoring its growing threat to global health [18, 19].

High vector competence and widespread human susceptibility have facilitated the continued expansion and adaptive evolution of CHIKV. Evidences from the 2014 outbreak in Latin America suggests that CHIKV may cause a greater burden than any other arthropod-borne virus [20, 21]. Advances in DNA sequencing and bioinformatics have enabled extensive viral genome surveillance, improving our understanding of the genetic diversity and transmission dynamics of emerging viruses [22]. In this study, we explored the epidemiology and evolution of CHIKV during its emergence using global whole genome sequences to further elucidate the genetic evolution and transmission potential of CHIKV.

## 2. Methods

### 2.1. Whole Genome Sequence Searching and Typing

As of November 13, 2023, CHIKV whole-genome sequences longer than 11 kb were downloaded from NCBI (https://www.ncbi.nlm.nih.gov/). Collection country, year, and host information were recorded (Supporting Information 1: Table S1). Sequences were uploaded to the CHIKV typing website (https://www.genomedetective.com/app/typingtool/chikungunya/) to obtain genotype information for all the sequences. Country distribution and time-series maps were visualized using the R package.

### 2.2. Host Composition and Vector Distribution

As of April 5, 2024, the worldwide distribution of *Ae. albopictus* and *Ae. aegypti* from 1953 to 2024 was obtained using GBIF (https://www.gbif.org/). Additional host information from NCBI sequences included *Homo sapiens* (*n* = 802), *Ae. aegypti* (*n* = 16), *Ae. albopictus* (*n* = 11), *Aedes furcifer* (*n* = 4), *Macaca fascicularis* (*n* = 4), *Aedes luteocephalus* (*n* = 2), *Culex quinquefasciatus* (*n* = 2), *Aedes dalzieli* (*n* = 1), *Aedes africanus* (*n* = 1), *Aedes opok* (*n* = 1), *Anopheles* (*Ceilia*) *funestus* (*n* = 1), sentinel mouse (*n* = 1), and Chiroptera (*n* = 1). Host composition was visualized using the R language.

### 2.3. Phylogenetic Analysis

A total of 929 whole genome sequences were aligned using MAFFT [23] and the aligned sequences were evaluated and edited in Aliview [24]. The evolutionary distances were computed using the optimal GTR + F+ R8 model, the phylogenetic tree was constructed with the maximum likelihood (ML) method. Bootstrap values are given for 1000 replicates. Phylogenetic trees were generated using IQTREE v2.3.5 [25] and visualized and annotated at iTOL [26] (https://itol.embl.de/). Outer rings were added to display genotype and host information, and branches were colored by country.

### 2.4. System Dynamics Reconstruction

The E1 gene of CHIKV is the basis for the classification of CHIKVs into three genotypes: Asian, WA, and ECSA, and its sequences are relatively conserved [7]. In addition, a single mutation in the E1 gene has previously caused a large-scale outbreak of CHIKV [27], and E1 sequences have been used for the phylogenetic analyses of CHIKV [28]. We selected the whole-genome sequences of the ECSA and Asian genotypes with available country and time information (~30% of each genotype), and all available WA sequences with such information (ECSA = 138, Asian = 119, and WA = 12; Supporting Information 2: Table S2). After alignment with MAFFT, E1 sequences were obtained for subsequent analysis using Aliview. To avoid the effects of recombination before assessing temporal signals and inferring time-scale phylogenies, recombination analyses were performed on the E1 of CHIKV to investigate potential recombination events using the Phi test in RDP4 [29], using RDP, GENECONV, Bootscan, Maxchi, Chimera, Si Sscan, Phylpro, LARD, and 3Seq to identify recombination events. Temporal signal detection was performed on nonrecombinant sequences to test the relationship between genetic diversity and sampling date, using TempEst [30] to regress the root tip genetic distances on the ML tree against the exact sampling date and to remove outlier sequences based on the estimated root tip distances. To obtain more robust rate estimates, the Bayesian Monte Carlo Markov chain (MCMC) method implemented in the BEAST software package v1.10.4 [31] was used to first select the optimal nucleotide model for the sequences based on the Bayesian information criterion (BIC) using the ModelFinder plug-in in the PhyloSuite [32], and later to run the file using the Beauti configuration. The uncorrected lognormal relaxation molecular clock model and the Bayesian Skyline merger tree prior were selected. Multiple runs of MCMC were merged using LogCombiner v1.10.4 with 200 million generations per group and sampling every 20,000 generations. Convergence of the relevant parameters was assessed using Tracer v1.6 [33] (effective sample sizes [ESSs] for relevant model parameters >200, few ESSs > 100) and skyline plots of the three genotypes were obtained. The posterior distributions of the trees obtained from the BEAST analysis were analyzed using TreeAnnotator v1.10.4 to obtain maximum spectral confidence (MCC) trees after having a 10% burnin. The MCC trees were visualized using FigTree v1.4.4 (http://tree.bio.ed.ac.uk/software/figtree/).

### 2.5. Spatial and Temporal Dynamic Analysis

To track the spatial and temporal dynamics of the three major CHIKV genotypes globally, a phylogeographic analysis was performed using SPREAD3 v0.9.7.1 [34] by applying an asymmetric substitution model of positional transformations and estimating positional diffusivity using the Bayesian stochastic search for variable selection (BSSVS) model, with the Bayes factor (BF) test selected to identify significant links between geographic regions. Circos plots and maps were used to visualize the probable routes. Circos plots were created using the Cnsknowall website (https://www.cnsknowall.com) and the maps were drawn by R packages.

### 2.6. Amino Acid Variation Analysis

To detect selection on all CHIKV proteins, coding region sequences were analyzed for recombination, and a total of 815 sequences were involved in the positive selection site analysis after removing recombinant sequences and sequences containing parsimonious and ambiguous bases. A ML tree based on available sequences was reconstructed using Datamonkey [35] (http://www. datamonkey.org/). Methods used to examine positive amino acid sites included single-likelihood-ratio ancestor counting (SLAC), fixed-effects likelihood ratio (FEL), evolutionary mixed-effects modeling (MEME), and fast unconstrained Bayesian inferential selection (FUBAR). The significance levels for SLAC, FEL, and MEME were set at a *p*-value threshold of 0.1, and the significance level for FUBAR was set at a *p*-value threshold of 0.9, we considered a site detected by more than two algorithms as positively selected, and the amino acid variability of the detected positively selected sites was visualized using SEQLOGO plots from the OmicShare (https://www.omicshare.com/tools). The Shannon entropy online analysis tool (http://www.hiv.lanl.gov/content/sequence/ENTROPY/entropy_one.html) was used to search for mutational hotspots in CHIKV, selecting nonconservative mutation sites with changes in the nature of amino acids among those with a Shannon entropy of more than 0.7, showing that mutations occurring in ≥20% mutations in the sequence. Amino acid sites with changes in amino acid hydrophobicity were selected among this sites and bar graphs were drawn using the R package. Homology modeling of consensus sequences for each protein was performed using the I-TASSER website [36] (https://zhanggroup.org/I-TASSER/), and protein models were visualized using UCSF ChimeraX [37].

## 3. Results

### 3.1. Spatial and Temporal Distribution of CHIKV

**Figure 1:**
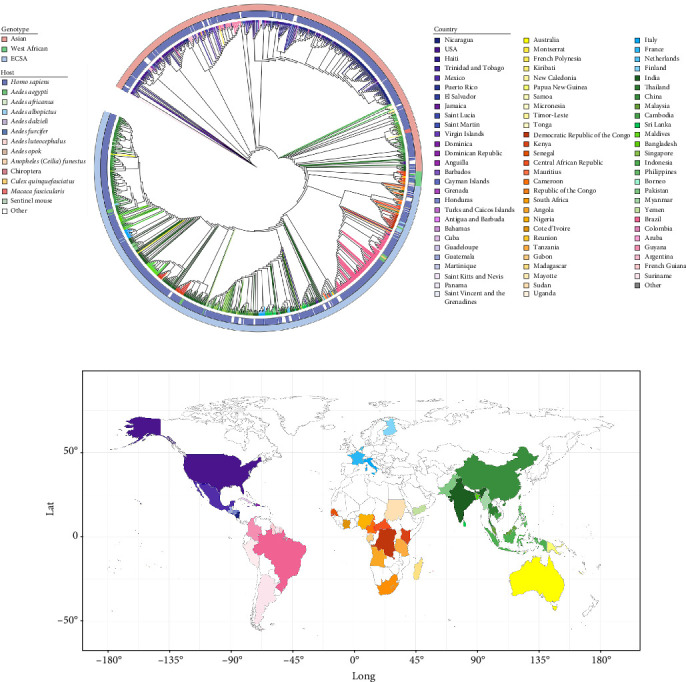
Geographic distribution of CHIKV whole genome sequences. (A) Maximum likelihood (ML) tree drawn based on 929 whole genome sequences of CHIKV. The inner circle in the outer ring of the evolutionary tree is the host distribution, the outer circle is the genotype distribution, and the color of the evolutionary branches in the ML tree corresponds to the countries of the sequences. (B) Distribution of the countries in which CHIKV whole genome sequences are located, and the color corresponds to the color of the branches in the ML tree.

We obtained 929 whole-genome sequences (>11 kb) from NCBI, of which 892 had collection dates: 12 WA, 485 ECSA, and 395 Asian genotypes (Supporting Information 1: Table S1). Plotting according to the collection time, it can be seen from Supporting Information 3: Figure S1 that after about 2005, the number of CHIKV sequences began to grow gradually, and there was a big outbreak of Asian genotypes in 2014, and two peaks of outbreaks of ECSA genotypes. In addition, a total of 912 sequences with national information were counted, including 14 WA, 500 ECSA, and 398 Asian genotypes. The whole-genome sequencing data of CHIKV now covers over 80 countries and regions worldwide, specifically, including 28 countries and regions in North America and the Caribbean, 18 countries in Africa, 15 countries in Asia, 10 countries in Oceania, 7 countries in South America, and 4 countries in Europe. Geographically, the sequences of Asian genotype are primarily found in the Americas, Asia, and Oceania, while the sequences of the WA genotype are mainly distributed in Africa. The sequences of the ECSA genotype are the most widespread and has become the dominant lineage circulating globally (Figure 1).

### 3.2. Host Composition and Transmission of CHIKV

**Figure 2:**
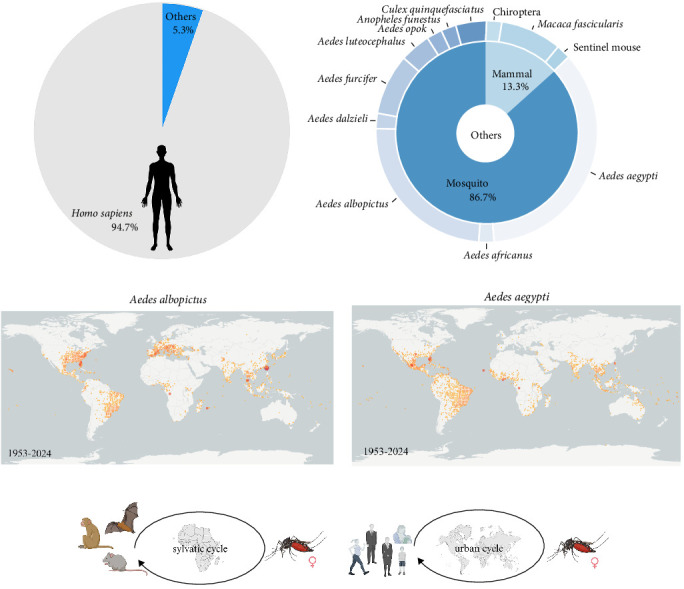
Host composition, distribution, and transmission of CHIKV. (A) Composition of human and non-human hosts of CHIKV based on host information of each sequence obtained from NCBI. (B) Detailed information on non-human hosts based on non-human host information. (C) Distribution of *Aedes albopictus* in the world in 1953–2024. (https://www.gbif.org/zh/occurrence/map?taxon_key=1651430&year=1953,2024). **(**D) Distribution of *Aedes aegypti* in the world in 1953–2024. (https://www.gbif.org/zh/occurrence/map?taxon_key=1651891&year=1953,2024). **(**E) Sylvatic and urban cycles of CHIKV (created with BioRender.com).

According to the statistics of the data downloaded from NCBI, it was found that about 94.7% of the hosts of CHIKV were humans (Figure 2A). Among the non-human hosts, mosquitoes accounted for a larger and more diverse proportion, with a total of nine species of mosquitoes, including seven species of *Aedes*, one *Anopheles*, and one *Culex* (Figure 2B). The host distribution of the three genotypes can be seen in the host exosphere of the evolutionary tree (Figure 1A), where the WA genotype has six hosts, including humans, mosquitoes, bats, and rats, which corresponds to the fact that the WA genotype is found mainly in the sylvatic cycle. The ECSA and the Asian genotypes are found mainly in the urban cycle, and the transmission is mainly sustained through the mosquito-human link. From the distribution of the two main vectors of CHIKV, *Ae. albopictus*, and *Ae. aegypti*, the two mosquito species are widely distributed in the world (Figures 1A and 2C, D), with a lesser distribution in the African region, which also confirms that the transmission of CHIKV in Africa mainly relies on the forest cycle (Figure 2E). The wide distribution of the main vectors also implies that CHIKV has the potential to spread worldwide.

### 3.3. Evolutionary Dynamics of Three Genotypes

E1 sequences intercepted from whole-genome sequences were analyzed phylogenetically on a time scale. The root-to-tip distance analysis revealed a positive correlation between genetic distance and sampling time in the selected datasets of the WA (*R*^2^ = 0.7192, correlation coefficient = 0.8481), ECSA (*R*^2^ = 0.6772, correlation coefficient = 0.8229), and Asian (*R*^2^ = 0.9099, correlation coefficient = 0.9539) genotypes. These findings accord with the molecular clock theory, thereby validating the dataset's appropriateness for phylogenetic molecular clock analysis (Supporting Information 4: Figure S2).

**Figure 3:**
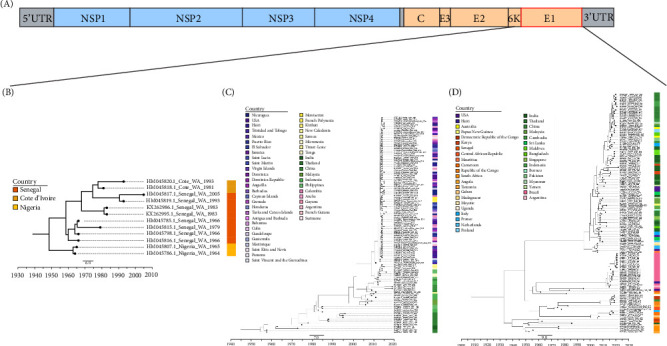
CHIKV three genotypes of MCC tree. (A) The genomic structure of CHIKV. (B) The MCC tree of the WA genotype. (C) The MCC tree of the Asian genotype. (D) The MCC tree of the ECSA genotype.

**Figure 4:**
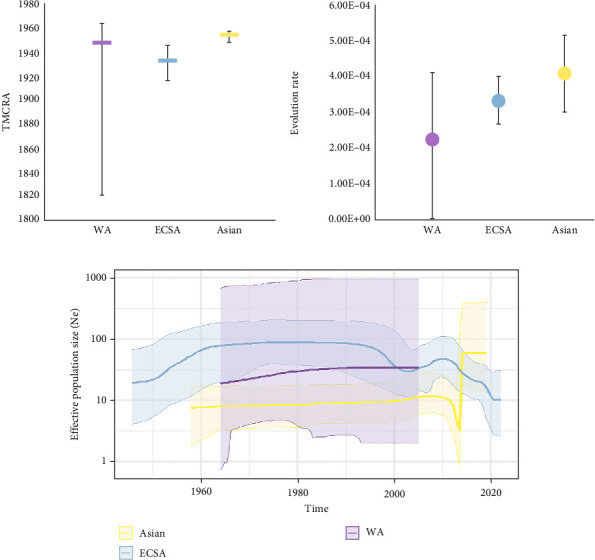
Comparison of evolutionary characteristics of the three major genotypes of CHIKV. (A) The mean time of the most recent ancestor (TMRCA) and 95% HPD interval for each genotype. (B) The mean evolutionary rate (substitutions per site per year, substitution/site/year) and 95% HPD interval for each cluster were estimated using an uncorrelated relaxed molecular clock model. (C) Effective population size was estimated by time and 95% HPD interval using the Skyline coalescent model. The logarithmic effective number of infections (Ne) was plotted against the viral generation time representing effective transmissions over time.

From the MCC trees obtained, it can be seen that the WA genotype originating strain was isolated from Nigeria with the mean time of the most recent ancestor (TMRCA) dating back to 1948 (95% HPD: 1820−1964), the ECSA genotype originating strain was isolated from Tanzania with the TMCRA dating back to 1933 (95% HPD: 1917–1946), and the Asian genotype originating strain was isolated from Thailand with the TMCRA dating back to November 1954 (95% HPD: October 1948–November 1957) (Figures 3 and 4A). Estimating the evolutionary rate and 95% HPD of the three major lineages with a relaxed molecular clock model, the evolutionary rate of the WA genotype was 2.23 × 10^−4^ (95% HPD: 1.09 × 10^−7^–4.11 × 10^−4^) substitutions/site/year, and the evolutionary rate of the ECSA genotype was 3.31 × 10^−4^ (95% HPD: 2.67 × 10^−4^–4.01 × 10^−4^) substitutions/site/year, and 4.09 × 10^−4^ (95% HPD: 3.01 × 10^−4^–5.16 × 10^−4^) substitutions/site/year for the Asian genotype, which could be seen that the Asian genotype had a higher evolutionary rate (Figure 4B). Using the Skyline model to estimate the effective population size by time and 95% HPD intervals, it can be seen that the population size of the ECSA genotype peaked around 1960 and then declined slightly around 1997 until it reached a second peak around 2010, which corresponds to the history of the ECSA genotype outbreaks. The Asian genotype peaked around 2014, while the WA genotype showed minimal fluctuation with wide confidence intervals (Figure 4C).

### 3.4. Phylogeography

**Figure 5:**
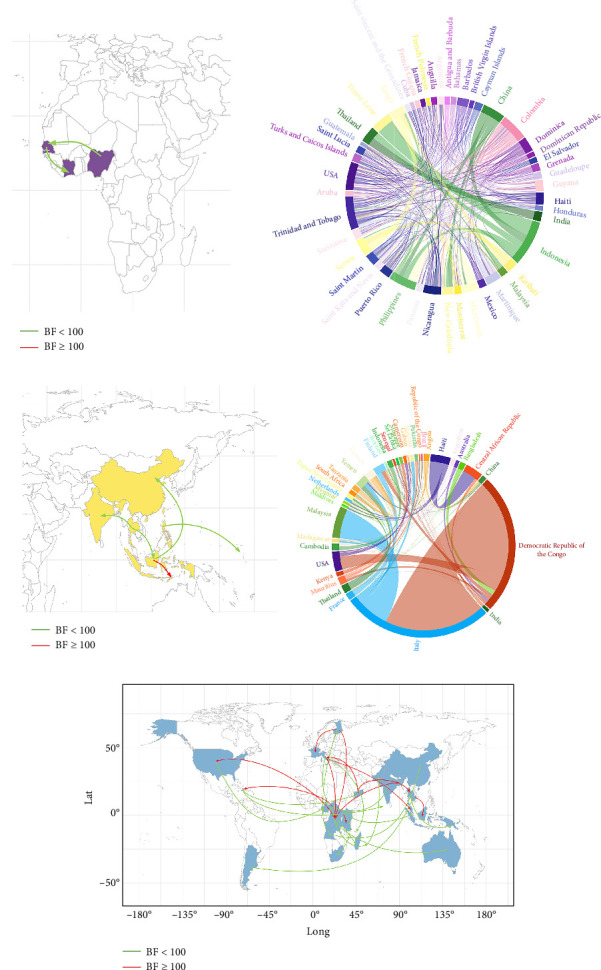
Circos plots and maps visualizing the transmission pathways of the three major genotypes of CHIKV. (A) Possible transmission pathways for the WA genotype (BF > 3, PP > 0.5). (B) Possible transmission pathways for Asian genotype (BF > 3). (C) Possible transmission pathways for Asin genotype (BF > 3, PP > 0.5). (D) Possible transmission pathways for ECSA genotype (BF > 3). (E) Possible transmission pathways for ECSA genotype (BF > 3, PP > 0.5). (The thickness of the lines in circos plots reflects the strength of support for virus movement (BF). Red lines in the map represent transmission pathways with BF > 100 and green lines represent transmission pathways with BF < 100).

Phylogeographic analysis of E1 gene sequences reconstructed 70 years transmission history for the three CHIKV lineages and explored their global spatiotemporal routes. A possible pathway was identified for the WA genotype, suggesting spread from Nigeria to Côte d'Ivoire, and finally to Senegal (Figure 5A). The Asian genotype originated in Thailand, and in terms of transmission, it spread mainly from the Asian region to the Oceania region before 2014, and then to the Americas and spread rapidly in the Americas after 2014, with localized circulations and a few spreads to Oceania. In addition, we also found that the Asian genotype in the St. Maarten region of the Caribbean may have existed before the outbreak of the epidemic from China or the Philippines to Trinidad and Tobago. However, according to the most possible transmission route obtained by BF > 3.0 and posterior probability (PP) > 0.5, we can see the activity of Asian genotype in Asia and Oceania, but there is no transmission route to the Americas. It is speculated that the results may be inaccurate because the datasets involved in the analysis are not complete (Figure 5B, C). The combination of the MCC tree and the circos plot shows that the ECSA genotype spread around the world, from Tanzania to African countries, spread widely among African countries, and then spreads to Asia, Europe, the Americas, and Oceania, with localized circulations between continents as well. The most probable transmission routes based on BF >3.0 and PP >0.5 show that the Democratic Republic of the Congo is an important hub for the spread of the CHIKV ECSA genotype from Africa, and Italy is an important hub for the spread of the genotype from Europe (Figure 5D, E).

### 3.5. Amino Acid Variation

**Figure 6:**
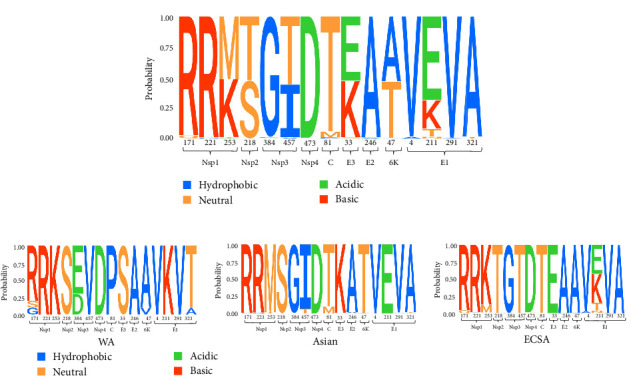
Amino acid variation at positively selected sites. (A) Amino acid variation at positively selected sites for all sequences of CHIKV. (B) Amino acid variation in sequences of the three genotypes of CHIKV.

**Figure 7:**
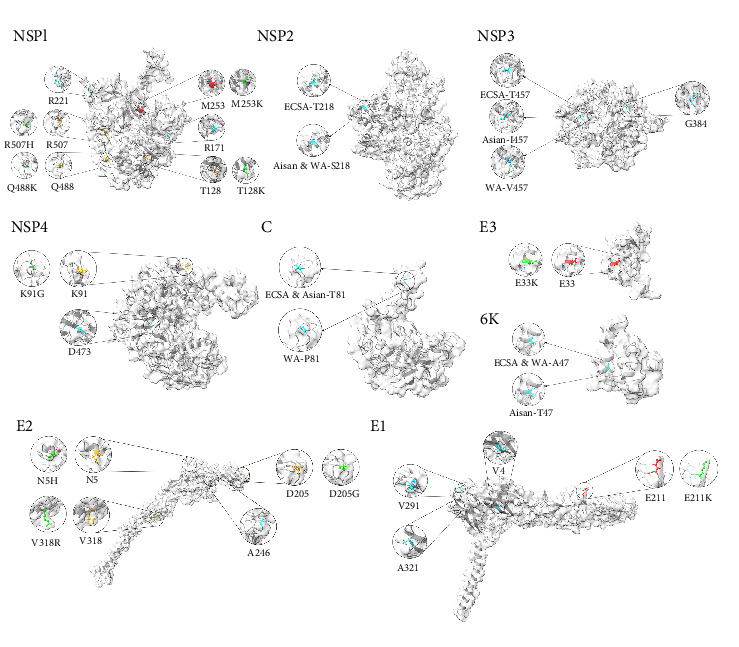
Structures of CHIKV proteins demonstrating positive selection sites, as well as highly mutated sites with altered hydrophobicity. (Blue sites represent positive selection sites, orange represents amino acids at the highly mutated site in the consensus sequence, red represents the site that is both highly mutated and positively selected, and green represents the mutated amino acids).

We performed selection pressure and mutation analysis of CHIKV. Detection of positively selected sites in individual proteins identified 15 codons subject to positive selection, with four sites subject to positive selection detected in the E1 protein, three in the NSP1 protein, two in the NSP3 protein, and one in each of the NSP2 protein, NSP4 protein, C protein, E3 protein, E2 protein, and 6K protein (Figure 6A and Table 1). A statistical analysis of the amino acid distribution of each positive selection site revealed that sites 253 in NSP1, 218 in NSP2, 457 in NSP3, 81 in C, 47 in 6K, and 211 in E1 showed variability across genotypes (Figure 6B and Supporting Information 5: Table S3). Shannon entropy analysis of individual proteins revealed variable sites in the sequences, and entropy values above the threshold of 0.7 indicated that these sites were highly variable (Supporting Information 6: Table S4). The amino acid hydrophobicity changed at the highly mutated sites of each protein were amino acids 128, 253, 488, and 507 of NSP1, 91 of NSP4, 33 of E3, 5, 205, and 318 of E2, and 211 of E1 (Figure 7). In the positive selection site analysis and Shannon entropy analysis of individual proteins, it was found that both the number of positive selection sites and the number of high mutation sites that undergo nonconservative mutations were higher in the NSP1 protein, the NSP3 protein had the highest number of high mutation sites that undergo nonconservative mutations, and the E1 protein had the highest number of positive selection sites. Among them, 253 in NSP1, 457 in NSP3, 33 in E3, 47 in 6K, and 211 in E1, which are all positive selection sites and high mutation sites that undergo nonconservative mutations.

## 4. Discussion

### 4.1. Transmission History of the Three Genotypes

Previous studies have considered the global evolution and spread of global CHIKV sequences across the globe [28]. In our study, the three genotypes of CHIKV were studied separately using phylogenetic, Bayesian, and phylogeographic analyses to better clarify the evolutionary and dispersal history of the three lineages. The evolutionary dynamics and spread of the ECSA genotype confirmed that the ECSA genotype is the dominant genotype, capable of spreading widely around the world, and that there is a tendency for the ECSA genotype to spread to non-endemic areas as human activities increase across geographical boundaries and as climate change occurs. However, the severe scarcity of genomic data for the WA genotype hinders a comprehensive elucidation of its transmission dynamics from the available sequences. Consequently, critical gaps persist, impeding both our understanding of and response to recurrent outbreaks. From the comparison of the evolutionary characteristics of the three major genotypes, it is evident that the Asian genotype has a high evolutionary rate and has shown an alarming range and speed of spread since it spread to the Americas, which confirms the pandemic potential of this genotype. The results of the study were also allowed to verify that the large-scale outbreak experienced in French Polynesia from October 2014 to January 2015 may have been introduced to French Polynesia from the Americas and not from other Pacific countries [38]. Our study identified hub countries for outward transmission of the ECSA genotype and the possibility that transmission of the Asian genotype to non-endemic areas of the world may be associated with travel cases and their indigenous transmission, which provides new ideas for putting in place controls for the re-emergence of CHIKV outbreaks.

### 4.2. Amino Acid Variation in Nonstructural Proteins

Given that a single substitution causes significant differences in the specificity and pathogenicity of the alphavirus vectors, we performed a selection pressure versus mutation analysis of CHIKV. CHIKV encodes four nonstructural proteins (NSP1, NSP2, NSP3, and NSP4) that are involved in a range of processes, including genome replication and immune regulation. Among the four viral nonstructural proteins, NSP1 is the only membrane-associated protein responsible for the localization of the viral replication complex and anchoring to the replication site on the membrane [39]. Amino acids 171, 221, and 253 in NSP1 are in positive selection, and amino acids 128, 253, 478, 488, and 507 in NSP1 are highly mutated sites. It has been demonstrated that the positively charged residue K253 in CHIKV NSP1 is required for interaction with negatively charged lipids in the membrane, and that the mutation and selection of this site has an important effect on the binding capacity of NSP1 [40]. Amino acid 211 in the positively selected state is located in one of the membrane-attached (MA) loops of NSP1, and the NSP1 protein is clustered by two intertwined MA loops, where positively charged residues form a cluster around the membrane insertion site and establish electrostatic interactions with phosphatidylserine lipids enriched in the inner leaflet of the plasma membrane, which are essential for RNA replication [41]. Amino acids 128 and 171 in NSP1 have not yet been studied to confirm their functional impact, in addition, the entire C-terminal tail after amino acid 474 in NSP1 is disordered, and we found three highly mutated amino acid sites in this range, whose functional impact remains to be investigated.

The NSP2 protein is a key protein for viral proliferation and possesses both RNA triphosphatase and helicase activities in its N-terminal domain [42]. The positive selection site 218 found in NSP2 is located in the N-terminal deconjugate enzyme structural domain of the NSP2 protein, where the amino acids of the ECSA genotype are different from those of the other two genotypes, and the function of this site may be related to deconjugate enzyme function [43].

The NSP3 protein has been recognized as essential for viral replication and adaptation to its host [44], and both the positive selection site and the hypermutation site found in NSP3 are located in the C-terminal hypervariable structural domain (HVD), which is disordered in NSP3 and serves as a platform for interactions with a wide range of host proteins. The linear motifs contained in the HVD of CHIKV NSP3 have been shown to recruit defined host protein families into the formation of functional viral replication complexes [45]. There is a direct link between CHIKV RNA synthesis and NSP3 phosphorylation, and elimination of phosphorylation sites in HVD inhibits CHIKV replicase activity [46]. Whereas the variants at the highly mutated loci identified in this study were all associated with threonine, the functional impact of these loci needs to be further verified.

The NSP4 is an RNA-dependent RNA polymerase (RdRp) that is a core subunit of the viral replication complex [47]. Amino acid 473 in NSP4 is a positive selection site found in NSP4, and positions 91, 107, and 506 are highly mutated. The N-terminal region of NSP4 (100 residues long) forms a partially unstructured structural domain, which is necessary for the proper functioning of NSP4, and can be targeted to block its polymerase activity, thus inhibiting viral replication in the host cell [48]. The C-terminal RdRp structural domain also plays a key role in catalyzing genomic RNA replication and transcription [49]. These sites identified in this study can be further investigated for these functions.

### 4.3. Amino Acid Variation in Structural Proteins

CHIKV contains five structural proteins, C, E3, E2, E1, and 6K proteins. The main function of the C protein is to form nuclear capsids capable of self-cleaving from structural polyproteins prior to genomic RNA binding [50]. Our study identified amino acid 81 as a positive selection site in the C protein, which is located in the N-terminal structural domain of C and is involved in genomic RNA encapsulation. Although the amino acid 81 is not a highly mutated site in the C protein, we found that the WA genotype differs from the other two genotypes at this site. It has been found that nucleolus localization sequences located between 60 and 99, which are involved in nuclear import, were identified in the N-terminal region of the C protein, mutations in the C protein NoLS have been shown to attenuate replication in mammalian and mosquito cells [51], but the functional impact of amino acid differences at this site remains to be verified.

The E1 and E2 glycoproteins are primarily responsible for membrane fusion and viral entry into host cells, where E2 interacts with and attaches to cellular receptors, and E1 is involved in fusion of viral and cellular cell membranes. The E1 protein contains three distinct *β*-structural domains, structural domains I, II and III (DI, DII, and DIII), and DII has a highly conserved hydrophobic fusion loop, which, when the pH of the endosome is lowered, it is exposed to the surrounding environment, triggering the E1 rearrangement and leading to the insertion of the hydrophobic fusion loop into the cell membrane. Our study found that amino acids 4, 211, 291, and 321 in E1 were under positive selection, with the 211 also being a high mutation site. The E1-K211E mutation has been shown to enhance the fitness of *Ae. aegypti* [52]. Amino acid 211 of E1 is the most variable among the ECSA strains, and in addition to the K211E mutation, it has been found that K211T plays a role in viral attachment to cells [53]. Amino acids 145 and 304 are also mutation sites with high entropy values, and no studies have yet demonstrated the functional impact of mutations at these sites. Among them, amino acid 145 is located in the DII domain, and it is worthwhile to investigate the effect of mutation at this position on the function of E1 for cell membrane fusion. The E2 protein also consists of three distinct structural domains, structural domains A, B, and C. The receptor binding site is located in structural domain A, whereas structural domain B is located in the outermost layer of each spicule and shields the fusion loop on structural domain II of E1, and structural domain C is located closest to the viral membrane [54]. In the E2 protein we found three highly mutated sites, sites 5, 205, and 318 and one site under positive selection, site 246. It can be seen from the 3D structure of the protein that 205 is located in structural domain B and 318 is located in structural domain C. The functional implications need to be further explored.

Compared with other proteins, the E3 and 6K proteins have been less studied. The E3 protein plays a central role in the regulation of the E2 protein folding and binding to the E2–E1 dimer [55]. In the E3 protein we found that amino acid 33 is both positively selected and highly mutated, and the amino acids at this site are different in all three genotypes. The 6K proteins are highly hydrophobic, cysteine-rich acylated proteins with a wide variety of functions, ranging from participation in envelope protein processing to membrane permeabilization, viral outgrowth, and viral assembly [56]. In the 6K protein we identified a positively selected site 47 that differed in the Asian genotype from the other two genotypes. These need to be further investigated to determine whether variation at this site affects the CHIKV phenotype.

### 4.4. Application Prospect of Amino Acid Variation Sites

The global spread of the three major genotypes of CHIKV confirms that the virus is expanding at a remarkable rate. Millions of people in tropical and subtropical regions are already affected or at high risk of infection, and countries with moderate climates may experience severe outbreaks due to the presence of mosquito vectors throughout the year. The global upsurge in CHIKV outbreaks and transmission is closely linked to the emergence of viral mutations that allow for increased viral adaptations to existing and novel vectors, the leading to increased transmission. Structural proteins encapsulate viral nucleic acids and coordinate viral particle assembly, and nonstructural proteins play key roles in viral replication, translation, and mediating host immune escape. Thus, both structural and nonstructural proteins are promising targets for the development of anti-CHIKV antiviral drugs. The first Chikungunya vaccine, Ixchiq, is now available, but it was developed based on the ECSA genotype and there are no specific antiviral drugs for CHIKV infection, which highlights the importance of monitoring the CHIKV genome [57]. We analyzed the selection pressure analysis and mutational hotspots of individual CHIKV proteins with the aim of providing new perspectives for the development of antiviral drugs for the treatment of CHIKV. The relevant sites obtained from the selection pressure analysis and mutation hotspot analysis of CHIKV proteins need to be further investigated by computational methods such as molecular dynamics and experimental methods, such as reverse genetics in order to clarify whether these sites can be targets for the control of CHIKV.

### 4.5. Limitations

Limitations of our study, where we based our statistical analyses only on CHIKV genome-wide data currently publicly available in NCBI, suggest that our analyses may have missed transmission events involving unsampled countries or countries that did not upload sequence information. For example, only 12 data containing country and temporal information were involved in the analysis for the WA genotype, and the lack of sampling resulted in the phylogenetic analysis of the WA genotype not clearly reflecting the phylogenetic results of the WA genotype, and also in the results obtained from phylogeographic analyses, the Nigerian and Côte d'Ivoire's were geographically closer to each other, but not predicting the paths of transmission between the two. In our phylogeographic analysis, we were unable to obtain specific sampling points for all sequences based on the information available, and then used the coordinates of each country's capital as the sampling location for the sequences. In addition, due to the limitation of computational resources, only part of the E1 gene sequences of CHIKV were sampled for the analysis of evolutionary dynamics and phylogeography in this study, and although we have tried our best to ensure that all the information of countries and time is included in the process of data selection, the final results are still deficient. Genomics studies can be combined with epidemiological data and vector distribution data to better track the spread and evolution of CHIKV, our study was limited by computational resources and limited publicly available information that prevented the results of our phylogeographic analyses from adequately demonstrating the spread of CHIKV, for example, our results lack the current prevalence of CHIKV in Brazil [58–60], which requires further data collection and analysis.

## 5. Conclusion

Our study, despite its limitations, was able to provide new insights into the origin, evolutionary dynamics, and global transmission pathways of CHIKV. The impact of CHIKF on human health has increased dramatically over the past two decades. Although its mortality rate is low, it has a high incidence of long-term disability, which constitutes a significant health risk [61]. As CHIKV continues to evolve and expand its geographic distribution, communication between continents has become more rapid and frequent. As CHIKV genome-wide data continues to be updated, we are better able to track the spread and evolution of CHIKV, and the potential discovery of new targeted control measures to help reduce its public health burden, through genomics studies. As the CHIKV genome-wide data continue to be updated, genomic studies will allow us to better track the spread and evolution of CHIKV and identify new targeted control measures. Combining genomic studies with epidemiological data (e.g., incidence rates, outbreak timelines, and demographic information) will allow us to more clearly define how genetic variation affects viral transmission patterns and public health, and thus identify new targeted control measures to help reduce its public health burden. In addition, we can explore new directions in drug and vaccine development based on positive selection or mutations during evolution.

## Figures and Tables

**Table 1:** Positively selected sites in proteins of CHIKV.

Protein	Site	MEME (*p*-value)	FEL (*p*-value)	SLAC (*p*-value)	FUBAR (Post. pro)
Nsp1	171	**0**	**0**	**0.000684**	**1**
	221	**0.01**	**0.0562**	0.157	0.861
	253	**0.04**	**0.0427**	**0.0804**	**0.969**

Nsp2	218	**0.09**	**0.0679**	0.205	0.826

Nsp3	384	**0.05**	**0.0384**	0.139	0.871
	457	**0.05**	**0.0376**	**0.089**	0.875

Nsp4	473	**0.02**	**0.0443**	0.233	**0.9**

C	81	**0.01**	**0.0737**	0.2	**0.953**

E3	33	0.1	**0.0934**	0.243	**0.941**

E2	246	**0.01**	**0.0197**	0.133	**0.908**

6K	47	**0.02**	**0.0212**	**0.0879**	**0.988**

E1	4	**0.04**	**0.093**	0.198	0.802
	211	**0.06**	0.122	0.167	**0.97**
	291	**0.01**	**0.0461**	**0.0878**	**0.947**
	321	**0.07**	**0.0488**	0.132	**0.905**

*Note:* Bolded values are those that meet the filter criteria.

## Data Availability

The datasets used and analyzed during the current study are included within the article.

## Supporting Information

Supporting Information 1 Table S1. Data on 929 complete CHIKV genomes obtained from NCBI (statistics through November 13, 2023), genotyping information was obtained from the CHIKV typing website (https://www.genomedetective.com/app/typingtool/chikungunya/).

Supporting Information 2 Table S2. Sampling data for Bayesian phylogenetic analysis.

Supporting Information 3 Figure S1. Temporal distribution of whole genome sequences of three CHIKV genotypes.

Supporting Information 4 Figure S2. Time-signal detection of three CHIKV genotypes.

Supporting Information 5 Table S3. Amino acid comparison of positively selected loci of CHIKV in three genotypes (number in parentheses is the number of sequences).

Supporting Information 6 Table S4. Highly mutated sites in proteins of CHIKV (the points marked in red are those where the hydrophobicity of the amino acid has changed, numbers in parentheses are sequence numbers).
