# Supplementary material for: Global Transmission and Evolution of Chikungunya Virus: Origins, Adaptive Mutations, and Intercontinental Spread of the Three Genotypes
Source: Transbound Emerg Dis. 2025 Nov 26;2025:3315650. doi: 10.1155/tbed/3315650 (PMC12674863; doi:10.1155/tbed/3315650)
Supplement: Supporting Information 3 — Figure S1. Temporal distribution of whole genome sequences of three CHIKV genotypes. [file 3315650.f3.docx]

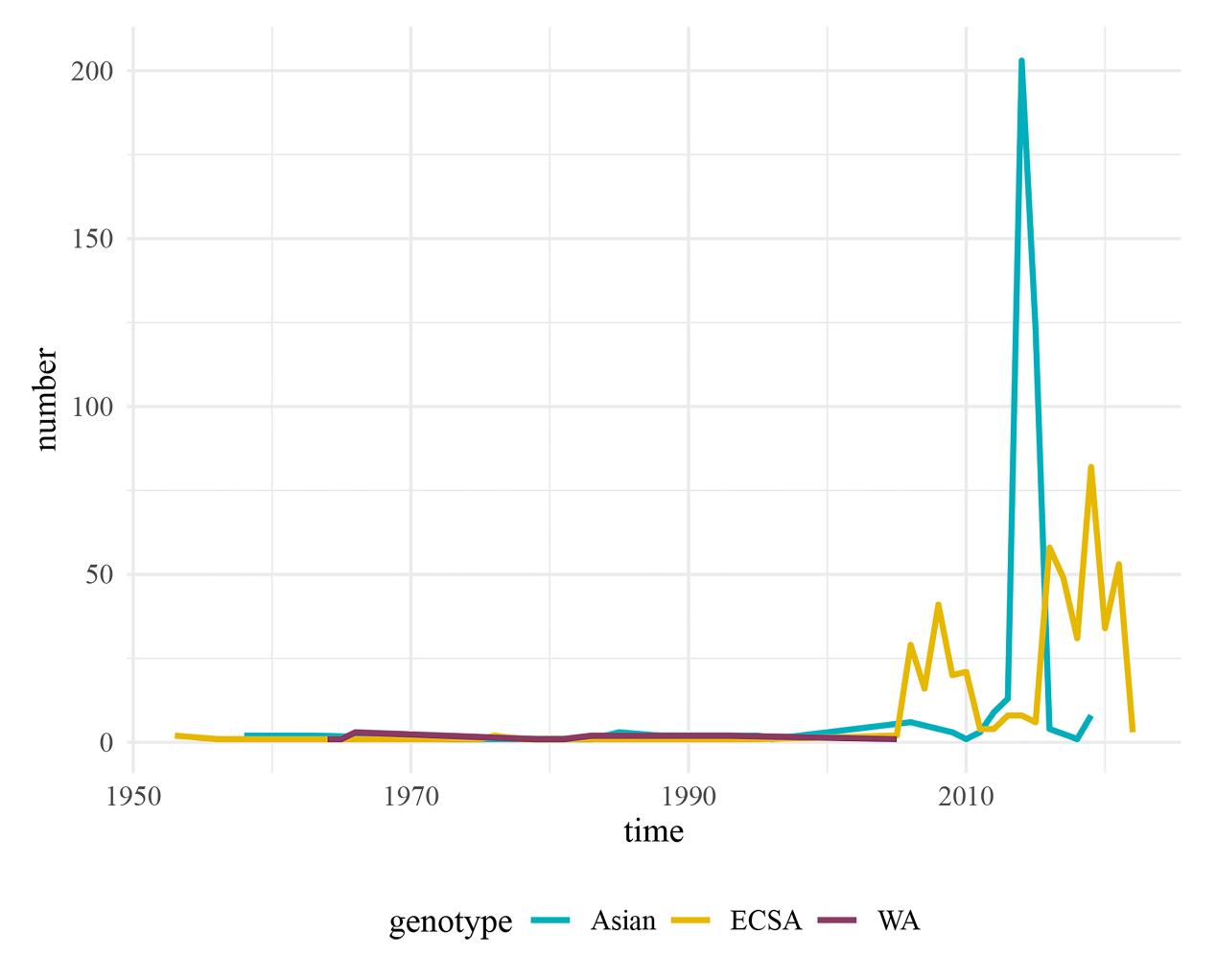


**Supplementary figure 1. Temporal distribution of whole genome sequences of three CHIKV genotypes.**
