# Supplementary figures and images for: Global Transmission and Evolution of Chikungunya Virus: Origins, Adaptive Mutations, and Intercontinental Spread of the Three Genotypes
Source: Transbound Emerg Dis. 2025 Nov 26;2025:3315650. doi: 10.1155/tbed/3315650 (PMC12674863; doi:10.1155/tbed/3315650)

**
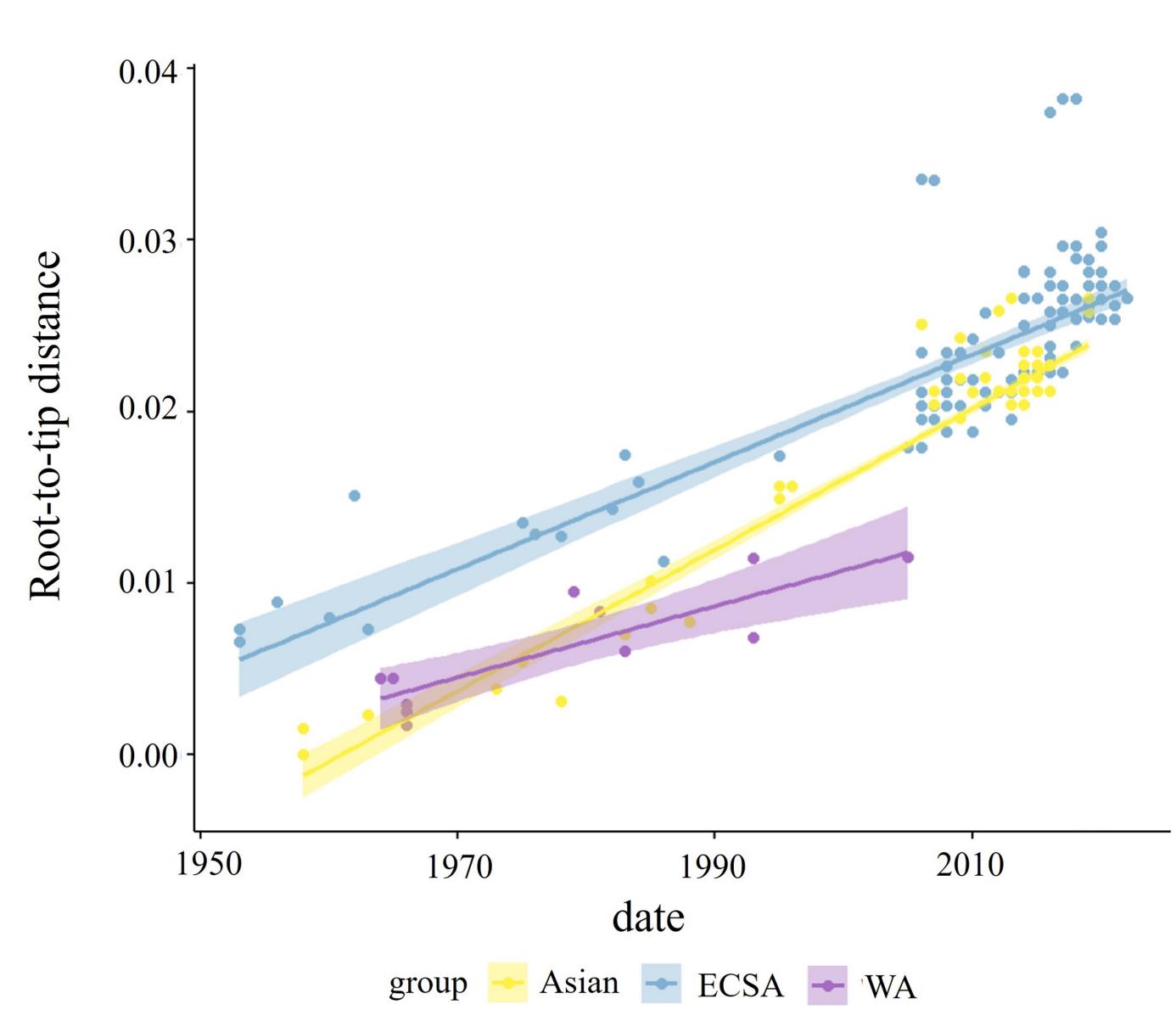
**

**Supplementary figure 2. Time-signal detection of three CHIKV genotypes.**

Supplement: Supporting Information 4 — Figure S2. Time-signal detection of three CHIKV genotypes. [file 3315650.f4.docx]
